# Artificial Intelligence and Emerging Digital Technologies Across the Stroke Continuum: From Risk Prediction to Real-Time Monitoring and Rapid Response

**DOI:** 10.3390/medicina62071254

**Published:** 2026-06-29

**Authors:** Matteo Gregorini, Lorenzo Lorusso, Larissa Airoldi, Maria Di Stefano, Anna Formenti, Gabriele Lucchi, Paola Melzi, Elisabetta Perego, Elena Tagliabue, Antonio Tetto, Manuela Vaccaro

**Affiliations:** 1 Institute of Informatics and Telematics (IIT-CNR), 56124 Pisa, Italy; 2 Neurology and Stroke Unit, Merate Hospital, ASST-Lecco, 23807 Merate, Italy; l.airoldi@asst-lecco.it (L.A.); m.distefano@asst-lecco.it (M.D.S.); a.formenti@asst-lecco.it (A.F.); ga.lucchi@asst-lecco.it (G.L.); el.perego@asst-lecco.it (E.P.); e.tagliabue@asst-lecco.it (E.T.); a.tetto@asst-lecco.it (A.T.); m.vaccaro@asst-lecco.it (M.V.)

**Keywords:** stroke prevention, AI, rapid response

## Abstract

Stroke remains a leading cause of death and long-term disability worldwide, making prevention strategies a global health priority. Emerging technologies—including artificial intelligence (AI), wearable devices, digital health applications, and drone-assisted emergency systems—are increasingly being explored to improve stroke prevention and early management. In primary prevention, machine learning models can identify individuals at high risk of stroke using clinical and behavioral data with high reported predictive accuracy, although most models are derived from retrospective, single-center datasets and still require prospective external validation. Digital devices and wearable technologies enable continuous monitoring of cardiovascular risk factors and support behavioral interventions aimed at reducing vascular risk. In secondary prevention, AI-based tools are being developed to predict stroke recurrence, identify modifiable risk factors, and detect patients at risk of poor medication adherence. In the acute setting, AI-assisted neuroimaging platforms are already integrated into clinical and telestroke workflows, supporting rapid triage and treatment decisions. In parallel, drone-based emergency systems may contribute to improved outcomes by reducing prehospital delays and facilitating telemedicine-based triage in remote or resource-limited settings, although current evidence is derived largely from out-of-hospital cardiac arrest pathways rather than stroke-specific trials. Although advanced neurotechnological systems capable of real-time neurophysiological monitoring and closed-loop neuromodulation exist in other neurological disorders, their role in stroke prevention remains largely theoretical. Overall, these technologies offer promising opportunities to reshape the continuum of stroke prevention and care, but further validation, integration into clinical workflows, and evidence of real-world effectiveness are required before widespread implementation.

## 1. Introduction

Stroke represents one of the most significant global health challenges, accounting for substantial mortality and long-term disability [1]. Despite advances in acute treatment, prevention remains the most effective strategy to reduce the burden of cerebrovascular disease. Traditionally, stroke prevention has been divided into primary prevention, which focuses on reducing the risk of a first cerebrovascular event, and secondary prevention, which aims to prevent recurrence after an initial stroke. Recent technological innovations are transforming how prevention strategies are conceptualized and implemented. Artificial intelligence (AI) and machine learning (ML) methods can analyze large clinical and population datasets to identify individuals at elevated risk of stroke and support personalized preventive strategies [2,3]. At the same time, wearable devices and mobile health technologies enable continuous monitoring of cardiovascular risk factors and promote behavioral interventions that address modifiable determinants of stroke risk [4,5,6]. In addition to predictive and monitoring technologies, emerging tools such as drone-based emergency systems are being investigated as a means to improve the timeliness of stroke care [7,8,9,10,11,12].

**Figure 1:**
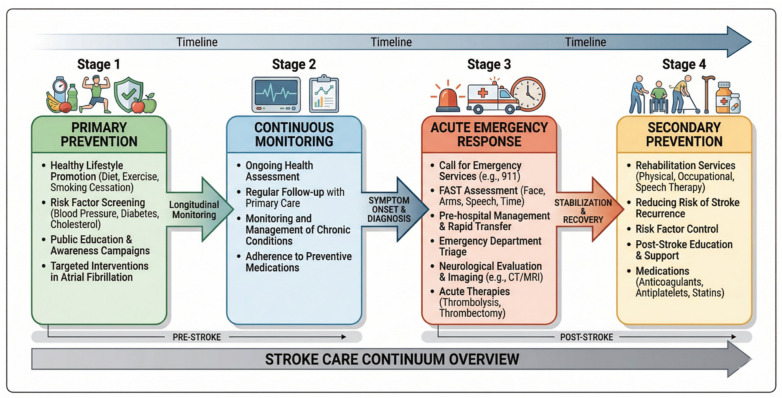
The stroke care continuum, from primary prevention and continuous monitoring to acute emergency response and secondary prevention.

Although these systems do not prevent stroke in the traditional sense, they may reduce the time between symptom onset and treatment, thereby limiting neurological damage. Taken together, these innovations are reshaping stroke care across a continuum that includes risk prediction, continuous monitoring, behavioral intervention, and rapid emergency response. This review summarizes current evidence on the role of artificial intelligence, digital devices, wearable technologies, and drone-assisted systems in stroke prevention and early management. The overall stroke care continuum is summarized in Figure 1.

This article is a narrative review. The relevant literature was identified through a structured search of PubMed/MEDLINE, Scopus, Web of Science, and IEEE Xplore, complemented by manual screening of the reference lists of retrieved articles. The search combined terms related to stroke (e.g., “stroke”, “cerebrovascular disease”, “ischemic stroke”) with terms describing the technologies of interest (e.g., “artificial intelligence”, “machine learning”, “deep learning”, “wearable devices”, “mobile health”, “digital health”, “neuroimaging”, “drones”, “unmanned aerial vehicles”, “mobile stroke unit”). Priority was given to peer-reviewed studies published in English between January 2015 and 2026, although a small number of earlier seminal references were retained for context. Articles were selected on the basis of their relevance to stroke prevention and acute care across the care continuum, methodological quality, and contribution to the topics addressed; given the narrative design, no formal quantitative synthesis or risk-of-bias assessment was performed.

## 2. Artificial Intelligence in Primary Stroke Prevention: Risk Stratification and Population Screening

Artificial intelligence has emerged as a promising tool for identifying individuals at increased risk of stroke before the occurrence of a first event [2,13,14]. Machine learning algorithms can analyze large datasets containing demographic, clinical, and behavioral variables—including age, blood pressure, diabetes status, smoking habits, body mass index, and cardiovascular history—to estimate individual stroke risk [14,15,16,17,18].

Several studies have demonstrated high predictive performance using machine learning approaches such as random forests, gradient boosting, and ensemble models [15,16,17,19,20,21,22,23]. Reported predictive accuracies often exceed 90%, with some studies reporting accuracy between 93% and 99% when using optimized ensemble algorithms [15,17,21,22,24]. These models can capture complex nonlinear relationships among risk factors that may not be fully captured by traditional statistical risk scores [13,14]. However, these figures should be interpreted with caution: many of the underlying studies are retrospective, single-center, or based on relatively small or imbalanced datasets, which—together with the risk of overfitting and limited external validation—can inflate apparent performance. Because reported accuracies are derived from heterogeneous datasets, populations, and validation strategies, they cannot be directly compared across studies, and prospective, multicenter validation is needed before these models can be reliably adopted in clinical practice.

Explainable artificial intelligence (XAI) techniques have also been introduced to improve the interpretability of predictive models. Methods such as SHAP and LIME enable identification of the most influential variables contributing to risk predictions [16,18,19,20,21,23,25,26]. Commonly identified predictors include age, hypertension, diabetes, body mass index, and cardiovascular disease history [13,14,15,16,17,18]. Improving model transparency is considered essential for clinical adoption, as clinicians must understand how predictions are generated before incorporating them into patient care [2,14,27]. Table 1 summarizes the main algorithm types, predictors, accuracy ranges, and explainability tools discussed in this section.

In addition to research applications, web-based tools and digital interfaces have been developed to allow clinicians and individuals to estimate stroke risk using machine learning-based models [15,19]. These platforms may facilitate early identification of high-risk individuals and support targeted preventive interventions [2,27,28].

## 3. Digital Devices and Wearables for Stroke Prevention: Mobile Applications and Behavioral Interventions

Digital health technologies are increasingly being used to support stroke prevention strategies, particularly by promoting healthier behaviors and improving adherence to preventive measures. Smartphone applications designed for stroke risk assessment and health education allow users to monitor risk factors and receive personalized recommendations related to lifestyle changes [29].

Pilot randomized controlled trials and controlled studies evaluating mobile technology-based interventions have demonstrated modest improvements in cardiovascular and behavioral health metrics, including blood pressure control, lipid profile, physical activity, and diet quality [5]. These applications typically focus on promoting smoking cessation, improving diet, increasing physical activity, and supporting adherence to preventive medications or secondary prevention regimens [5,30].

Mobile health strategies may also include structured text messaging systems, goal-setting tools, reminders, and semi-interactive self-management packages designed to reinforce healthy behaviors and self-monitoring [4,30]. While such interventions have been shown to improve preventive behaviors, medication adherence, and intermediate risk factors, their direct impact on stroke incidence or recurrence remains uncertain, with few trials using hard endpoints and mostly showing no significant differences in stroke events [29]. Further large, long-term studies are required to clarify effects on stroke incidence.

### 3.1. Wearable Technologies and Continuous Monitoring

**Figure 2:**
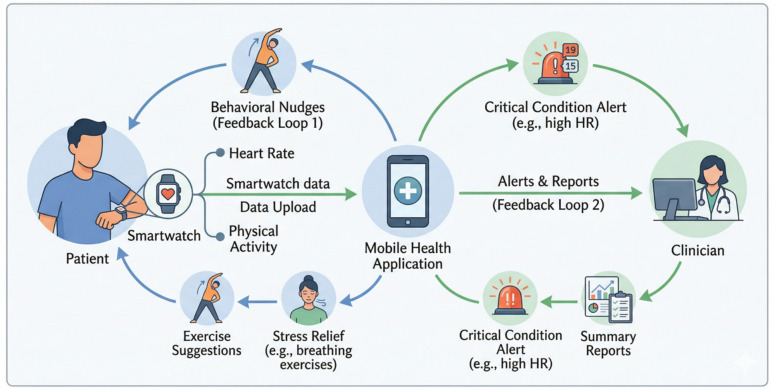
The digital health ecosystem linking wearable data capture, mobile health applications, clinician alerts, reports, and behavioral feedback loops. Arrows indicate the direction of data flow and feedback between components; Feedback Loop 1 returns behavioral nudges to the patient, and Feedback Loop 2 delivers alerts and reports to the clinician.

Wearable devices such as smartwatches, activity trackers, and other biosensor-based systems (as shown in Figure 2) allow continuous monitoring of physiological parameters relevant to stroke risk [5,29,31]. These devices can track variables such as heart rate, physical activity levels, sleep patterns, and—in some cases—subclinical atrial fibrillation [5].

Continuous monitoring may allow earlier detection of cardiovascular abnormalities and provide opportunities for timely intervention, particularly for intermittent conditions such as paroxysmal atrial fibrillation [32,33]. In particular, wearable technologies capable of detecting atrial fibrillation have attracted significant interest because atrial fibrillation is a major risk factor for ischemic stroke [31,34,35].

Although wearable devices can increase awareness of cardiovascular risk and encourage healthier behavior, direct evidence demonstrating reduction in stroke incidence remains limited; even intensive AF screening with continuous monitoring has not yet shown a clear stroke reduction in randomized trials [29,35]. Their most realistic role at present is therefore as tools for risk-factor monitoring and patient engagement rather than as stand-alone preventive interventions [29,31,35].

### 3.2. Real-Time Physiological and Neurophysiological Monitoring

Real-time physiological and neurophysiological monitoring relies on advanced neurotechnologies that can sense and modulate neural activity using closed-loop control, but current clinical uses focus on symptom control in other disorders rather than stroke prevention. Recent advances in neurotechnology have enabled real-time monitoring of electrophysiological and neurochemical signals within the nervous system. In certain neurological disorders, such as movement disorders and epilepsy, adaptive closed-loop neuromodulation systems are capable of dynamically adjusting stimulation parameters based on real-time physiological feedback. Examples include adaptive deep brain stimulation and phase-dependent neuromodulation systems that modify neural stimulation in response to ongoing neural activity. These systems demonstrate the technical feasibility of closed-loop neural monitoring and regulation.

However, such technologies are not currently used for stroke prevention. Most existing systems are invasive and primarily designed for symptom control rather than modification of vascular risk. As a result, although real-time neurophysiological modulation represents an important technological frontier, its application in stroke prevention remains speculative. Current clinical practice therefore relies primarily on wearable devices, smartphone applications, and digital monitoring tools aimed at identifying and modifying cardiovascular risk factors rather than directly regulating neural activity [5,29]. Looking ahead, the same closed-loop principles could in theory be extended to stroke—for example, to continuously detect cerebral ischemia or neurophysiological markers of recurrence risk and trigger timely intervention—but such applications remain hypothetical and would require substantial miniaturization, non-invasive sensing, and rigorous clinical validation before they could contribute to stroke prevention.

## 4. Artificial Intelligence in Secondary Stroke Prevention: Predicting Stroke Recurrence

Preventing recurrent ischemic stroke is a key objective of post-stroke management [36,37]. Machine learning models have been developed to predict recurrence using combinations of clinical data, laboratory variables, imaging features, and large registry datasets [36,38,39].

Several studies report predictive performance with area under the curve (AUC) values around 0.70–0.80 for recurrence prediction across different time horizons. Abedi and colleagues reported AUROC values from 0.69 to 0.79 for 1- to 5-year prediction windows [36]. Vodencarevic and co-workers achieved an AUC of 0.70 for 1-year recurrence using registry data [38], while Colangelo and colleagues reported AUCs of 0.71–0.76 for early and long-term recurrence in the PRERISK model [37]. Models based on radiomics and multimodal data have achieved AUCs up to about 0.79–0.86 [39].

These models often identify age, body mass index, glycated hemoglobin, lipid levels, renal function markers, and other vascular risk factors as key predictors of recurrence [36,37,39]. Large-scale registry studies involving tens to hundreds of thousands of patients have enabled development of more robust models capable of predicting recurrence during hospitalization or in long-term follow-up periods [38,40].

### 4.1. Personalized Risk Scores and Clinical Decision Support

Recent work has focused on integrating machine learning-based recurrence predictors into clinical decision-support tools capable of generating individualized recurrence risk estimates [36,37,38,39]. For example, AI-based platforms such as PRERISK estimate the probability of stroke recurrence at multiple time points, including 90 days, one year, and longer follow-up periods, and compare machine learning models with traditional Cox regression [36,37].

These systems can also simulate how improved control of modifiable risk factors (e.g., blood pressure, lipids, diabetes, smoking, BMI) would reduce recurrence risk over time, providing dynamic curves that change with different levels of risk-factor control [37]. Several models are implemented as web calculators or visual interfaces to support real-time clinical use and patient counseling [38,39,41].

Such tools have the potential to support more personalized follow-up strategies, targeted monitoring, and tailored secondary prevention in high-risk subgroups (e.g., symptomatic intracranial stenosis, minor ischemic stroke) and to improve communication between clinicians and patients regarding preventive interventions [40].

### 4.2. Predicting Medication Adherence

Medication adherence plays a critical role in secondary stroke prevention. Machine learning models are increasingly being used to identify patients at risk of poor adherence following hospital discharge.

Early predictive models demonstrate high recall for detecting individuals likely to discontinue or poorly adhere to preventive medications. For example, the PREDICT-POOR_COMP tool for post-stroke patients achieved recall around 0.9 for predicting poor compliance at 90 days after discharge, both in internal and external validation [42]. Decision-tree and other machine learning models applied to stroke screening or cardiovascular risk cohorts also show good discrimination for low adherence, supporting their use to flag high-risk patients.

Identifying such patients early may enable targeted interventions such as telemonitoring programs, patient education, or digital reminders aimed at improving adherence. Systematic reviews and meta-analyses show that remote and mHealth interventions can significantly improve medication adherence and related risk-factor control after stroke.

## 5. Drone-Assisted Technologies for Prehospital Stroke Care: Reducing Prehospital Delays

Stroke is a time-dependent medical emergency in which early diagnosis and treatment are essential to minimize brain injury. Rapid access to imaging, thrombolysis, or mechanical thrombectomy significantly improves clinical outcomes.

Unmanned aerial vehicles (UAVs), commonly referred to as drones, are being explored as potential tools to reduce prehospital delays in emergency medicine [7,43,44]. These systems have been studied for rapid delivery of critical medical equipment such as automated external defibrillators, blood products, and emergency medications, showing substantial time savings compared with conventional emergency medical services, particularly in rural or hard-to-reach areas [8,10,45,46,47].

In the context of stroke, drone-based systems could potentially support remote or rural healthcare settings by delivering critical supplies, transporting laboratory samples, or assisting emergency teams in reaching patients more quickly, thereby bridging delays between symptom onset and definitive care [7,12,48]. Although direct stroke-specific drone trials are limited, evidence from other time-critical emergencies suggests that integrating drones into prehospital networks may help reduce prehospital delays and improve access to advanced stroke treatments [7,43,44].

### 5.1. Telemedicine and Remote Triage

Drones equipped with cameras and communication systems may also facilitate telemedicine-based patient evaluation in remote or difficult-to-reach environment [43,49]. Such systems could allow healthcare professionals to remotely assess patients and guide triage decisions toward appropriate stroke centers. Aerial imagery and real-time video enable preliminary triage, identification of needs, and situational awareness prior to the arrival of responders, reducing the time required for locating victims and initiating treatment in challenging scenarios such as mountainous areas or disaster zones [7,8]. This approach may be particularly useful in regions with limited healthcare infrastructure or in situations where traditional emergency medical services face logistical challenges [49].

### 5.2. Mobile Stroke Units: Bringing the Hospital to the Patient

In parallel to emerging experimental prehospital technologies, Mobile Stroke Units (MSU) represent a highly effective, non-drone prehospital system innovation with mature clinical evidence. Stroke is a time-dependent medical emergency where early diagnosis and treatment are essential to minimize brain injury. To bridge the gap between symptom onset and definitive care, MSUs have been deployed as specialized ambulances equipped with onboard computed tomography (CT) scanners, point-of-care laboratory testing, and telemedicine capabilities. This technological setup effectively brings the hospital’s acute stroke diagnostic capabilities directly to the scene of the emergency. The primary advantage of the MSU is its ability to radically reduce the time to treatment. By performing neuroimaging directly in the transport vehicle, medical personnel can immediately rule out intracranial hemorrhage and identify ischemic events before the patient even reaches the hospital doors. Crucially, this allows for the administration of acute therapies, such as intravenous thrombolysis, directly within the ambulance. Initiating this treatment in the prehospital setting bypasses the traditional, time-consuming delays associated with emergency department triage, transportation to radiology, and waiting for laboratory results. Unlike drone-assisted emergency systems, which currently lack direct evidence for improved stroke outcomes and remain largely experimental, MSUs serve as a proven benchmark for outcome-oriented care. High-quality randomized controlled trials and robust observational data have consistently demonstrated that prehospital stroke evaluation and treatment via MSUs result in significantly faster thrombolysis metrics compared to standard emergency medical services. More importantly, these reduced treatment delays translate into tangible clinical benefits, showing improved long-term functional outcomes for patients who receive MSU care versus usual care. Consequently, MSUs underscore the immense potential of integrating advanced imaging and immediate treatment capabilities into the prehospital stroke care continuum.

### 5.3. Current Limitations

Despite promising conceptual models, drone-assisted emergency medical systems remain largely experimental. Most studies evaluating drone use in healthcare have focused on feasibility, modeling, or simulation rather than real-world, patient-centered clinical outcomes, and—where real-life deployments exist—they are largely outside stroke care (e.g., drone-delivered AEDs in OHCA) rather than stroke-specific response pathways [50,51,52]. Key challenges include regulatory barriers, safety considerations, integration with existing emergency medical services workflows, and public acceptance, and major guideline/consensus efforts continue to highlight the need for outcome-focused real-world research before recommending routine implementation of novel systems (including nonhuman resources) in time-critical emergencies. Moreover, there is currently little direct evidence demonstrating improved stroke outcomes associated with drone-based emergency response; by contrast, non-drone prehospital system innovations with mature clinical evidence—such as Mobile Stroke Units—have shown improved functional outcomes and faster thrombolysis metrics in randomized and high-quality observational data, underscoring the current evidence gap specific to drones in stroke systems of care [53,54,55]. Table 2 compares drones, mobile stroke units, and conventional emergency medical services across applications, advantages, and evidence gaps.

## 6. Artificial Intelligence in Acute Stroke Neuroimaging and Telestroke Decision Support

Beyond risk prediction and prevention, artificial intelligence has reached its most mature, real-world clinical implementation in the acute phase of stroke care, where AI-assisted neuroimaging platforms are routinely used to accelerate diagnosis and treatment decisions. Several commercially available, regulatory-approved decision-support systems—including Brainomix e-Stroke (Brainomix Ltd., Oxford, UK), RAPID (iSchemaView, Inc., Menlo Park, CA, USA), and Viz.ai (Viz.ai, Inc., San Francisco, CA, USA)—are now embedded in clinical and telestroke workflows in many comprehensive and primary stroke centers. These platforms automatically process non-contrast computed tomography (CT), CT angiography (CTA), and CT perfusion (CTP) images within minutes and return standardized, quantitative outputs that support rapid triage, thrombolysis and thrombectomy eligibility decisions, and inter-hospital transfer.

On non-contrast CT, automated tools such as e-ASPECTS generate the Alberta Stroke Program Early CT Score (ASPECTS) and flag early ischemic changes and intracranial hemorrhage, providing reproducible estimates that can assist less experienced readers; external validation studies have shown good agreement with expert consensus while highlighting variability in sensitivity across populations [56]. CT perfusion software such as RAPID automatically estimates the ischemic core and salvageable penumbra, and this approach underpinned patient selection in the DAWN and DEFUSE 3 trials that extended the thrombectomy time window [57]. On CTA, AI algorithms (e.g., Viz.ai) detect large-vessel occlusions and issue automated alerts to the stroke team; a systematic review and meta-analysis reported high diagnostic performance and significant improvements in workflow metrics, including reduced door-to-treatment and transfer times [58]. By coupling these outputs with cloud-based image sharing and communication tools, such systems are particularly valuable in telestroke and hub-and-spoke networks, where they help non-specialist sites identify candidates for reperfusion therapy and coordinate timely transfer.

Despite this relatively advanced level of adoption, important caveats remain. Reported performance varies with scanner type, image quality, and the characteristics of the validation population, and most evidence still derives from retrospective or single-vendor analyses; accordingly, these tools should augment rather than replace expert image interpretation. As with AI-assisted cardiovascular imaging, where comparable automated quantification techniques are advancing but still require methodological standardization and prospective validation before routine clinical translation [59], wider deployment of AI neuroimaging in stroke will depend on standardized acquisition and reporting protocols, transparent reporting of dataset heterogeneity, and prospective multicenter studies demonstrating improvements in patient-centered outcomes.

## 7. Discussion

Emerging technologies are transforming stroke prevention and management by introducing new approaches across the entire continuum of care. Artificial intelligence provides powerful tools for identifying individuals at risk and predicting recurrence after ischemic stroke. Digital devices and wearable technologies enable continuous monitoring of risk factors and promote behavioral interventions that may reduce long-term vascular risk. At the same time, technologies such as drone-assisted emergency systems offer innovative solutions for improving rapid access to care during acute stroke events. Although these systems do not prevent stroke in a traditional sense, they may contribute to improved outcomes by reducing delays in diagnosis and treatment; however, current real-world drone evidence largely comes from out-of-hospital cardiac arrest AED delivery rather than stroke-specific pathways [50,52]. Importantly, these technologies differ substantially in their level of clinical maturity. AI-based risk prediction models and wearable monitoring systems are approaching practical implementation, whereas drone-assisted stroke pathways remain in earlier stages of development. Future research should focus on validating predictive models in diverse populations, integrating digital technologies into existing healthcare systems, and evaluating whether these innovations can reduce stroke incidence and improve long-term outcomes; in parallel, mature prehospital system innovations such as Mobile Stroke Units provide a benchmark for outcome-oriented evaluation, having shown improved functional outcomes and faster thrombolysis metrics versus usual care [53,54,55].

Realizing the potential of these technologies will also require addressing important ethical, legal, and organizational challenges. AI-driven stroke prediction and imaging systems rely on large volumes of sensitive health data, raising concerns about data privacy, security, informed consent, and compliance with regulatory frameworks. Models trained on non-representative datasets may encode algorithmic bias and perform unequally across age, sex, ethnic, or socioeconomic groups, potentially widening rather than narrowing health disparities. Transparent reporting, external and prospective validation, continuous post-deployment monitoring, clear accountability for clinical decisions, and methodological standardization—mirroring efforts already underway in AI-assisted cardiovascular imaging and digital medicine [59]—will be essential to ensure that these tools are safe, equitable, and trustworthy. Equally important is meaningful clinical integration: technologies must fit existing workflows, demonstrate cost-effectiveness, and gain the trust of clinicians and patients before they can be adopted at scale.

## 8. Conclusions

Artificial intelligence and emerging digital technologies are reshaping the landscape of stroke prevention and early management. Machine learning models offer powerful tools for risk stratification and prediction of stroke recurrence, while digital devices and wearable technologies support continuous monitoring of modifiable risk factors and promote preventive behaviors. Complementary innovations such as drone-assisted emergency response systems may improve the timeliness of acute stroke care, particularly in underserved or remote regions. Although many of these technologies remain in developmental stages, their integration into coordinated stroke care pathways may ultimately contribute to more personalized, proactive, and efficient prevention strategies; however, current real-world drone evidence largely comes from out-of-hospital cardiac arrest AED delivery rather than stroke-specific pathways [50,51,52]. Further large-scale studies and real-world implementation trials will be essential to determine whether these technological innovations translate into measurable reductions in stroke incidence and improved patient outcomes; in parallel, Mobile Stroke Units provide a benchmark for outcome-oriented prehospital innovation in stroke, with evidence of improved functional outcomes and faster thrombolysis metrics compared with usual care [53,54,55].

## Figures and Tables

**Table 1:** Summary of AI-based stroke risk prediction models and explainability approaches. Reported predictive accuracies originate from heterogeneous datasets, study populations, and validation strategies and therefore cannot be directly compared across studies.

Algorithm Types	Key Risk Predictors Analyzed	Reported Predictive Accuracy	Explainability Tools (XAI) Used
Random forests	Age, blood pressure, diabetes status, smoking habits	Often exceed 90%	SHAP
Gradient boosting	Body mass index (BMI)	93–99% (when using optimized ensemble algorithms)	LIME
Ensemble models	Cardiovascular disease history	High ability to capture complex nonlinear relationships	Feature importance mapping for clinical trust and transparency

**Table 2:** Comparison of drone-assisted systems, mobile stroke units, and conventional emergency medical services in prehospital stroke care.

Modality	Primary Stroke Application	Key Advantages	Current Limitations & Evidence Gaps
Drones (UAVs)	Rapid delivery of medical equipment (AEDs, blood products) and telemedicine-based triage.	Substantial time savings compared to conventional EMS; reaches rural, remote, or hard-to-reach areas.	Largely experimental; faces regulatory barriers and safety considerations; lacks direct evidence for improved stroke outcomes.
Mobile Stroke Units (MSUs)	Prehospital stroke evaluation and treatment.	Serves as a benchmark with mature clinical evidence; shows faster thrombolysis metrics and improved functional outcomes compared to usual care.	Not explicitly detailed in text, but contrasted against drones as the proven standard for outcome-oriented care.
Conventional EMS	Standard emergency response and patient transport.	Ubiquitous baseline for standard care comparison.	Slower response times in rural/hard-to-reach areas compared to drones; faces logistical challenges in remote or disaster environments.

## Data Availability

No new data were created or analyzed in this study. Data sharing is not applicable to this article.
